# Characterization of Melanoma Cell Lines Resistant to Vemurafenib and Evaluation of Their Responsiveness to EGFR- and MET-Inhibitor Treatment

**DOI:** 10.3390/ijms21010113

**Published:** 2019-12-23

**Authors:** Ewelina Dratkiewicz, Aleksandra Simiczyjew, Katarzyna Pietraszek-Gremplewicz, Justyna Mazurkiewicz, Dorota Nowak

**Affiliations:** Department of Cell Pathology, Faculty of Biotechnology, University of Wroclaw, 50-383 Wroclaw, Poland

**Keywords:** melanoma, BRAF, vemurafenib, drug resistance, EGFR, MET, invasion, foretinib, lapatinib, cancer stem cells

## Abstract

Constitutively active mutated BRAF kinase occurs in more than 40% of patients suffering from melanoma. To block its activity, a specific inhibitor, vemurafenib, is applied as a therapy. Unfortunately, patients develop resistance to this drug rather quickly. Previously, we demonstrated that pairs of inhibitors directed against EGFR (epidermal growth factor receptor) and MET (hepatocyte growth factor receptor) trigger a synergistic cytotoxic effect in human melanoma cells, and decrease their invasive abilities. In this study, we aimed to generate and characterize melanoma cells resistant to vemurafenib treatment, and then to evaluate the effectiveness of a previously developed therapy in this model. We showed that melanoma cells resistant to the BRAF inhibitor are characterized by a lower proliferation rate and they acquire a spindle-like shape. Using Western Blot, we also noticed increased levels of EGFR, MET, and selected markers of cancer stem cells in generated cell lines. Resistant cells also exhibited increased invasive abilities and elevated proteolytic activity, observed using scratch wound assays and gelatin zymography. Moreover, combination therapy reduced their viability, as measured with a colorimetric cytotoxicity test, and decreased invasiveness. The obtained results validate the application of combination therapy directed against EGFR and MET in melanoma cells resistant to treatment with inhibitors of mutated BRAF.

## 1. Introduction

Among all patients suffering from skin cancers, only 4% are diagnosed with malignant melanoma. However, in this group melanoma is accountable for 80% of skin cancer-related deaths, mainly due to its high rate of metastasis [1]. In the last few years, a great number of molecular mechanisms responsible for the aggressiveness of this particular tumor have been identified, including somatic mutations and overexpression of genes encoding proteins involved in signal transduction pathways. One of the most prevalent drivers in melanoma progression is mutated BRAF, identified in more than 40% of patients suffering from this cancer [2,3]. BRAF is a member of the RAF serine-threonine family of kinases, which are components of the MAPK (mitogen-activated protein kinase) signaling pathway. They become active following the phosphorylation of their upstream effectors, RTKs (receptor tyrosine kinases). Upon ligand binding, RTKs undergo autophosphorylation and then recruit and activate RAS, which in turn phosphorylates RAFs [4]. The signal is then transduced to MEK1/2, followed by ERK1/2, which consecutively regulates their many substrates involved in substantial cell functions like proliferation or migration [5]. The majority of *BRAF* mutations occur in exon 15 at position 600, resulting in the substitution of valine for glutamic acid (V600E, 70–90%) or lysine (V600K, 10–30%). This aberration produces kinase, which is constitutively active independently of upstream regulators [6]. Fortunately, small molecule inhibitors directed against mutant BRAF have been developed and approved for use. Vemurafenib (PLX4032), a potent inhibitor of BRAF V600E that is recommended for cases of late-stage melanoma, prolonged patients’ overall survival from 9.9 to 13.2 months compared to standard chemotherapy [7]. However, signs of cancer progression can be detected within several months of the first administration of therapy, as a result of developed drug resistance. The resistance mechanisms include hyperactivation and overexpression of RTKs, reactivation of the MAPK pathway, hyperactivation of the PI3K (phosphoinositide 3-kinase)/AKT (protein kinase B) pathway, and changes in the cells’ interactions with the tumor microenvironment [8]. To combat emerging resistance to BRAF inhibitors, novel combination therapies have been developed, among which a treatment using inhibitors of BRAF and MEK, a downstream effector of BRAF, has shown the greatest potential so far [9].

In this study, we aimed to extend our previous work, where we tested a combination therapy directed against proteins frequently overexpressed in melanoma—EGFR (epidermal growth factor receptor) and MET (hepatocyte growth factor receptor)—in a panel of human melanoma cell lines and samples derived from patients. We obtained a synergistic cytotoxic effect in these lines, and observed a significant decrease in their invasive abilities upon inhibitor treatment [10,11]. To further examine the efficacy of the developed therapy, we generated cell lines resistant to vemurafenib treatment. Herein, we present a characterization of the established cell lines and their resistance mechanisms, which comprise the overexpression and hyperactivation of EGFR and MET, the emergence of cancer stem-like cell traits, and elevated invasive abilities. We also propose the dual inhibition of EGFR and MET as a potential therapy to overcome BRAF inhibitor resistance. 

## 2. Results

### 2.1. Establishing the Resistant Melanoma Cell Lines

**Figure 1:**
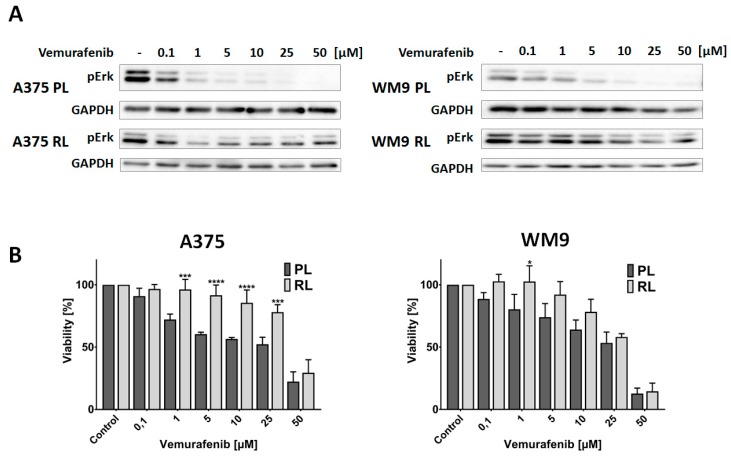
The sensitivity of parental and resistant cell lines to vemurafenib. (**A**) Inhibition of ERK phosphorylation in parental (PL) and resistant (RL) lines was evaluated using the Western Blot method. GAPDH was used as a loading control. Representative results of at least three experiments are shown. (**B**) Cell viability of parental (PL) and resistant (RL) lines was measured by an XTT assay following treatment with indicated concentrations of vemurafenib. The data represent the mean viability of three independent measurements ± SD. Asterisks indicate statistical significance vs. PL at * ≤0.05, *** ≤0.001, **** ≤0.0001.

Two human melanoma cell lines, derived from a primary amelanotic tumor —A375, and from metastasis to lymph nodes —WM9, were positively verified for the presence of BRAF V600E mutation. To check their sensitivity to vemurafenib, a selective inhibitor of mutated BRAF, Western Blot analysis and a cytotoxicity assay were performed. The obtained results show that the A375 cell line is more responsive to vemurafenib treatment compared to WM9, both in terms of the inhibition of phosphorylation of ERK kinase, which is a direct downstream effector of BRAF, and a decrease in viability (Figure 1A,B). Following the characterization of parental lines (PL), we started the establishment of cell lines resistant (RL) to vemurafenib. To achieve this goal, we cultured A375 and WM9 cells in the presence of increasing concentrations of BRAF V600E inhibitor, starting from 0.05 µM and doubling the amount of drug every two weeks. To verify if the cells had acquired resistance to vemurafenib, we conducted experiments analogous to the ones performed on parental cell lines. The collected results show that both cell lines exhibit resistance even to high concentrations of the used drug, seen as a prevalence of ERK phosphorylation and an increased cell viability (Figure 1A,B). A375 RL seems to demonstrate a higher level of resistance in terms of vemurafenib-mediated cytotoxicity, which can be also noticed in IC50 values for vemurafenib: 39.378 for the resistant line vs. 13.217 µM for the parental line (Figure S1). In the case of WM9 cells, these values were similar for both cell lines (ca. 20 µM).

### 2.2. Molecular and Morphological Changes of Generated Resistant Cells

**Figure 2:**
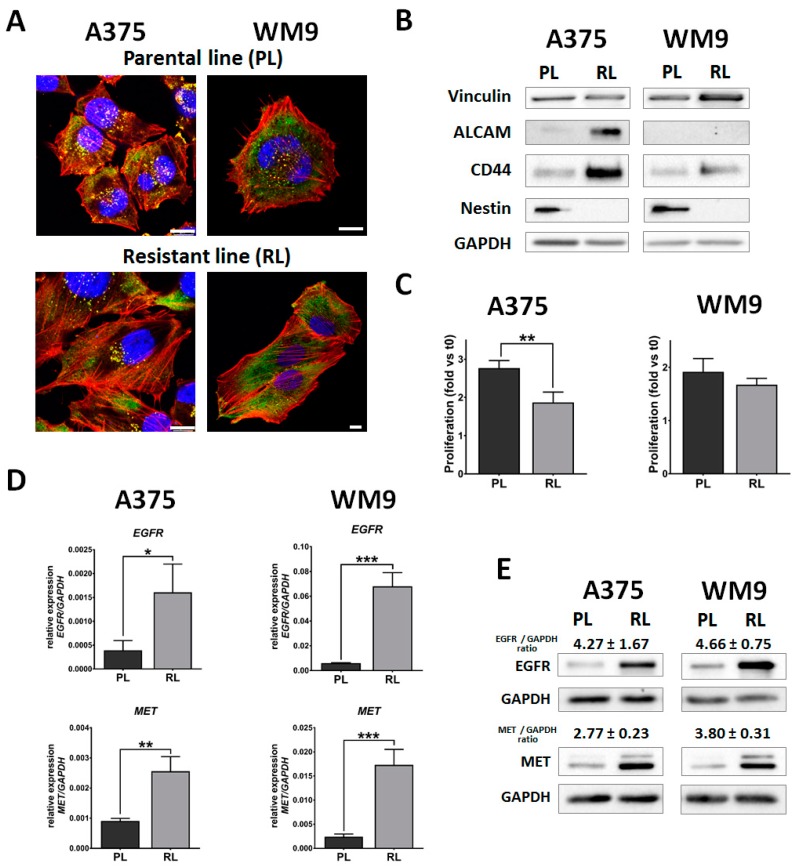
Characterization of resistant cells. (**A**) Morphology of parental (PL) and resistant (RL) cells seeded on Matrigel-coated cover slips visualized with confocal microscopy. Representative merged pictures of DNA (blue), F-actin (red) and cortactin (green) are shown. The scale bar is set at 10 µm. (**B**) Evaluation of vinculin, activated leukocyte cell adhesion molecule (ALCAM), CD44, and nestin levels using the Western Blot method, where GAPDH served as a loading control. Representative results of at least three experiments are shown. (**C**) Proliferation rate after 24 h measured with an XTT assay. The results are presented as the mean proliferation ± SD of three experiments, calculated in relation to the t0 plate. (**D**) The expression level of epidermal growth factor receptor (EGFR) and hepatocyte growth factor receptor (MET) evaluated with qPCR. Data represent the mean expression of three independent experiments relative to GAPDH ± SD. (**E**) Protein level of EGFR and MET determined with Western Blot, where GAPDH served as a loading control. Representative blotting membranes of three experiments are shown, accompanied by densitometry presented as a mean fold change ± SD of EGFR and MET versus GAPDH between parental and resistant lines. Asterisks indicate statistical significance at * ≤0.05, ** ≤0.01, *** ≤0.001.

Predictably, acquiring resistance to drug treatment, especially one targeting the element of the RAS/MAPK pathway responsible for elementary cell functions, entails a great number of changes in cells’ molecular biology and morphology. The first change noticed with a phase-contrast microscope was a different morphology of the resistant compared to the parental lines (Figure S2). To examine this feature in more detail, we employed immunofluorescent staining. Fixed cells were stained to visualize DNA (blue), cytoskeleton (F-actin, red) and the actin-associated protein cortactin (green), constituting the marker of invadopodia-adhesive structures with proteolytic activity, involved in cancer cell invasion (Figure 2A). We observed that resistant cells were more spread out, presented a spindle-like shape, and displayed highly pronounced stress fibers compared to the parental cells, while invadopodia were present in all examined cell lines. Elevated spreading of cells could be associated with changes in levels of focal adhesion proteins. For this reason, we checked one of the adhesive molecules, vinculin, and discovered that it was upregulated in WM9 RL (Figure 2B). The observation of altered morphology prompted us to check the markers of cancer stem-like cells (CSCs), which are characterized a.o. by a spindle-like shape. We noticed that resistant cells demonstrated elevated levels of some CSC markers. Both lines exhibited upregulation of CD44, a hyaluronic acid receptor, while an elevated level of CD166 (ALCAM, activated leukocyte cell adhesion molecule), was present only in A375 RL (Figure 2B). It was expressed neither in the parental nor in the resistant WM9 line. Rather surprising was the fact that both A375 RL and WM9 RL were deprived of nestin protein, an intermediate filament found in neural stem cells and many types of malignancies (Figure 2B) [12]. Additionally, we evaluated the proliferation rate of resistant cell lines and discovered that obtained cells, especially A375 RL, demonstrated reduced growth compared to parental lines, which adds to the CSC phenotype; however, in the case of WM9 RL this occurrence was not statistically significant (Figure 2C).

Due to the fact that cancer cells resistant to treatment often display overexpression of growth factor receptors, we checked the level of EGFR and MET in generated cell lines. We observed that both A375 RL and WM9 RL are discerned by a significant increase in the expression of EGFR and MET on mRNA and in the protein level (Figure 2D and E). A375 RL experienced a two- to fourfold increase in both receptors’ level, while for WM9 RL the change factor was even greater.

### 2.3. Invasive Abilities of Cells Resistant to Vemurafenib

Cancer cells resistant to drug treatment often demonstrate increased invasive abilities [13,14,15]. To evaluate the motility of resistant cells, we employed two assays: a spontaneous migration/invasion test, where there is no chemoattractant or directionality during cells’ movement; and a scratch wound assay, where cells move to close the wound formed on a confluent cell layer. In order to mimic the physiological niche of the melanoma cells, we used Matrigel, a mixture of extracellular matrix (ECM) proteins that imitates the composition of the basement membrane, on which the melanocytes and primary melanoma cells reside *in vivo*. For migration tests (standard 2D), we seeded cells to wells coated with Matrigel. To generate the conditions in which invasion tests were performed, we covered the cells seeded on the Matrigel coating with an additional ECM layer.

**Figure 3:**
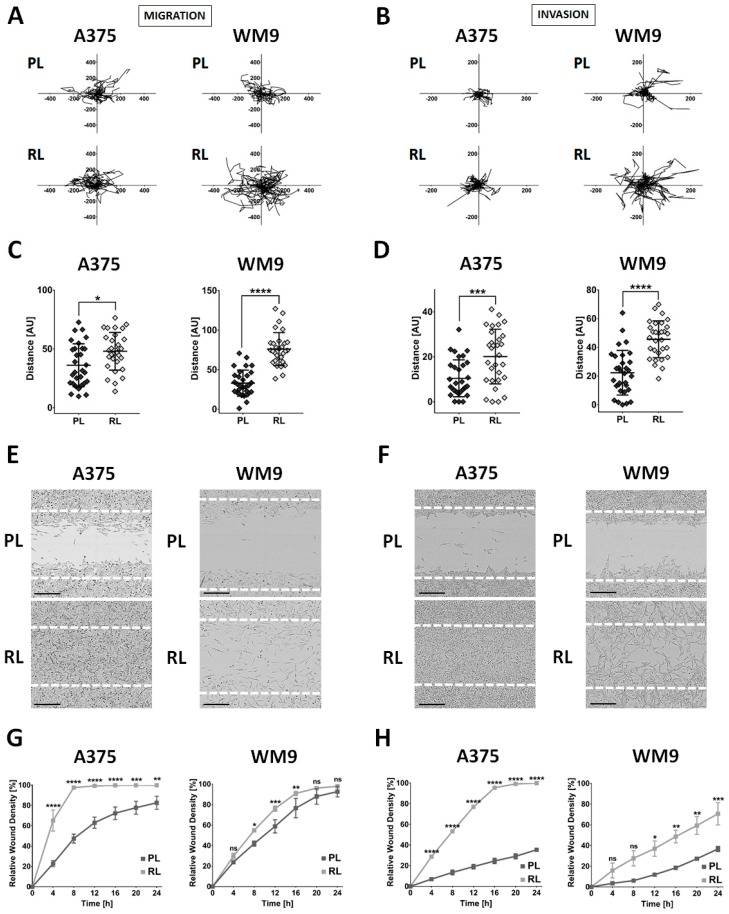
Invasive abilities of resistant cells. The ability of cells to spontaneously migrate was evaluated using the IncuCyte Live-Cell Imaging System with an interval of 2 h, where parental (PL) and resistant (RL) cells were seeded exclusively on a Matrigel layer or were covered with an additional Matrigel layer on top of them. Trajectories of migrating (**A**,**C**,**E**,**G**) or invading (**B**,**D**,**F**,**H**) cells are visualized with spider graphs. Distances (**C**,**D**) covered by cells in 24 h ± SD. At least 30 cells were quantified per repetition. The rate of migration (**E,G**) and invasion (**F**,**H**) of examined cells was assessed using a scratch wound assay, where cells migrate to close the wound. Representative images (**E**,**F**) of wounds covered by cells in 24 h are shown with white dotted lines indicating the edge of the wound at t0. The scale bar is set at 300 µm. Quantification (**G**,**H**) of the results is presented as the mean relative wound density obtained from three independent experiments ± SD. Asterisks indicate statistical significance at * ≤0.05, ** ≤0.01, *** ≤0.001, **** ≤0.0001, ns (not significant).

First, we tracked the spontaneous movement of the cells, using the IncuCyte Live-Cell Imaging System, and ImageJ software with the Manual Cell Tracking plugin. Based on the generated spider graphs and quantified distances covered by cells, we observed that resistant cell lines exhibited an increased rate of spontaneous migration (Figure 3A,C) and invasion (Figure 3B,D). In the case of A375 RL, this occurrence was more pronounced for cells embedded in the ECM, while WM9 RL exhibited double the rate of the parental line’s migration and invasion. Next, we performed wound healing analysis. Again, we noticed that cells resistant to vemurafenib migrated (Figure 3E,G) and invaded (Figure 3F,H) much faster compared to parental lines. During this assay, A375 RL showed the greatest increase in the rate of migration and invasion, as resistant cells in standard 2D conditions required only 6–8 h to completely cover the wound, while the parental cells were not able to realize this goal even after 24 h. Moreover, the elevated motile abilities of WM9 RL were more noticeable in the case of the invasion test.

### 2.4. Proteolytic Activity of Resistant Cells

While migration in two-dimensional conditions relies mainly on the contractile force generated by the actomyosin cytoskeleton and the turnover of focal adhesions, invasion additionally highly depends on the pathways generated in a dense extracellular matrix, along which the cells can travel. To facilitate this process, cells secrete matrix metalloproteases (MMPs), which are able to digest the proteins present in the microenvironment of cells [16]. 

**Figure 4:**
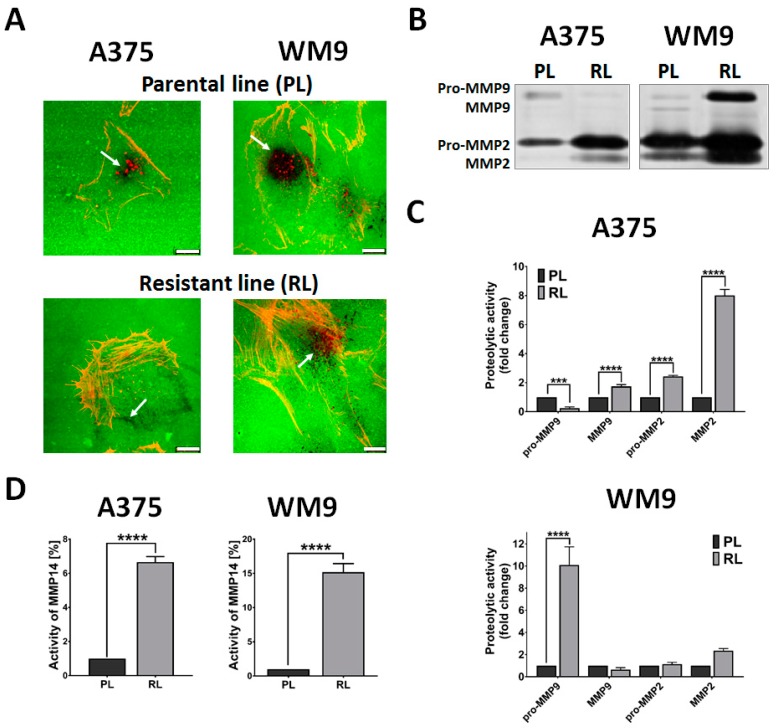
Proteolytic activity of resistant cells. (**A**) Parental (PL) and resistant (RL) cells were seeded on cover slips coated with FITC-conjugated gelatin (green) and visualized using phalloidin-Alexa 568 (F-actin, red). Arrows indicate the areas of proteolytic digestion. The scale bar is set at 10 µm. (**B**) Negative image of representative gelatin zymography gel. (**C**) Densitometry quantification of at least three independent repetitions of gelatin zymography. Results were normalized to total protein content. (**D**) Activity of MMP14 was measured in cell lysates using a fluorimetric activity assay. The data represent the mean MMP14 activity of three independent measurements ± SD. Asterisks indicate significance at *** ≤0.001, **** ≤0.0001.

Based on the previous results showing that resistant cells are characterized by an elevated rate of invasion, we verified whether they also exhibited increased proteolytic activity. First, to evaluate the pattern of digestion, we seeded the cells on cover slips coated with fluorescently-labeled gelatin (Figure 4A). The dark spots on the green background indicate the places of digestion by gelatinases. While A375 and WM9 parental lines showed sphere-like digestion patterns, in the case of resistant cells, separated dark spots (WM9 RL) or barely visible digestion along the edges of the cell (A375 RL) could be detected (Figure 4A). In the next step, we conducted a gelatin zymography assay using concentrated cell-conditioned media to establish which secreted gelatinases are more active in resistant cells (Figure 4B,C). While both resistant cell lines demonstrated an elevated level of pro- and active MMP2, in the case of WM9 RL, albeit not in statistically significant way, they differed regarding MMP9. Whereas the WM9 RL displayed a massive increase in pro-MMP9 rate with slight downregulation of active MMP9, an increase in the active MMP9 level with a simultaneous decreasing tendency of the pro-MMP9 rate was observed in the case of the A375 resistant cell line, which could be a result of MMPs’ compensation. However, it should be noted that the elevated level of MMP9 in A375 RL is not clearly seen on the zymography gel, while the densitometry followed by statistical analysis indicates significant upregulation of this metalloprotease.

Nevertheless, there are other metalloproteases that are able to digest the surrounding matrix. One of the most prominent enzymes in this group is MMP14 (also known as MT1-MMP), a collagenase which mainly digests type I collagen. To assess the activity of MMP14, we utilized a fluorimetric assay using whole cell lysates (Figure 4D). We observed a substantial increase in the activity of this protease in both resistant and parental cells, with fold change corresponding to values witnessed for the gelatinases. 

### 2.5. The Sensitivity of Resistant Cells to EGFR and MET Inhibitors

**Figure 5:**
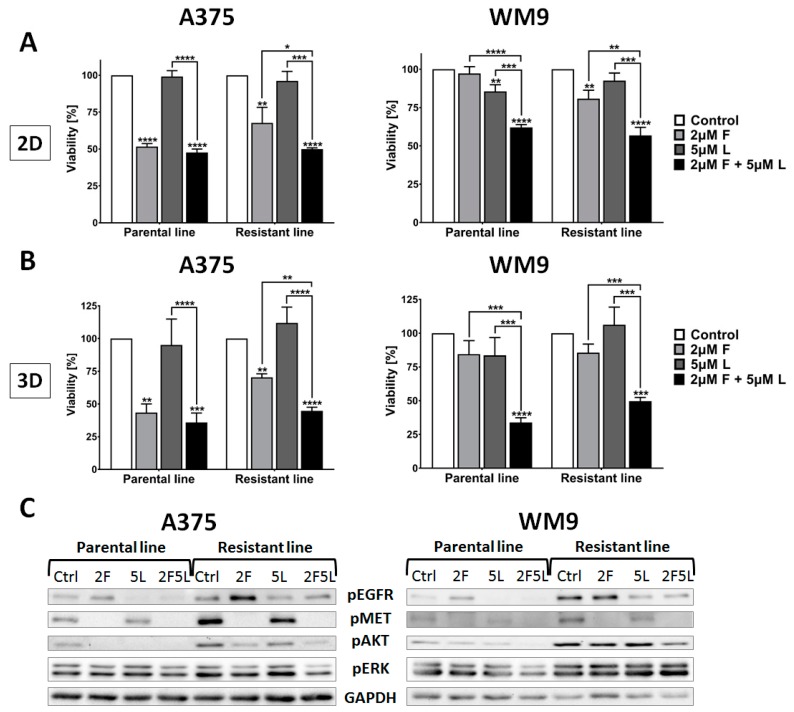
The sensitivity of resistant cells to EGFR and MET inhibitors. Cells were seeded (**A**) exclusively on a layer of Matrigel or (**B**) were covered with an additional layer of the extracellular matrix (ECM) on top of the cells and treated for 24 h with inhibitors of EGFR (L, lapatinib), and MET (F, foretinib) independently or as a pair at the indicated concentrations. Cell viability was evaluated using an XTT assay in (**A**) 2D and (**B**) 3D conditions. The results are shown as a mean value from three independent experiments ± SD. (**C**) Representative membranes from Western Blot analysis of mediators of signal transduction pathways inhibition after 4 h treatment with inhibitors of EGFR and MET. The membranes were probed against pEGFR, pMET, pAKT, and pERK. GAPDH was used as a loading control. Asterisks indicate statistical significance vs. control, unless indicated otherwise, at * ≤0.05, ** ≤0.01, *** ≤0.001, **** ≤0.0001.

As we have previously shown, EGFR and MET seem to be promising targets for anti-melanoma combination therapy using small molecule inhibitors. Foretinib (F, MET inhibitor) and lapatinib (L, EGFR inhibitor) used simultaneously were able to synergistically decrease the viability of melanoma cells, and also significantly diminish the invasive abilities and proteolytic activity of these cells [10,11]. Here, we have demonstrated that resistant cells exhibit overexpression of both studied receptors, which also corresponds to the elevated rate of migration and invasion. For this reason, we verified the sensitivity of established cell lines resistant to vemurafenib treatment, for inhibitors of EGFR (5 µM lapatinib) and MET (2 µM foretinib) used independently, and as a pair. First, we examined viability in 2D and 3D conditions, and we noticed that parental and resistant cells responded to the inhibitors’ treatment almost to the same extent in both setups (Figure 5A,B). Similarly to their respective parental lines, WM9 RL showed a significant decrease only after incubation with the pair of inhibitors, while A375 RL reacted to the pair of inhibitors and also to foretinib alone, albeit to a lesser extent. Considering that proliferation and protection against apoptosis are governed by signaling pathways such as MAPK and PI3K/AKT, which include constitutively active BRAF V600E, we looked into the activation status of its up- and downstream effectors. We performed Western Blot analysis using cell lysates collected after 4 h of cell incubation in the presence of EGF and HGF, ligands of EGFR and MET, respectively, and inhibitors of these RTKs (Figure 5C). Firstly, we noticed that both resistant cell lines exhibited hyperativation of EGFR, MET, and AKT, while phosphorylation of ERK was only slightly affected. A combination of foretinib and lapatinib treatment was sufficient to completely eliminate the activation of upregulated MET and EGFR in A375 RL, and even noticeably or slightly reduce the phosphorylation of AKT and ERK, respectively, compared to monotherapy. WM9 RL displayed slightly diminished sensitivity to drug treatment; however, only a pair of inhibitors was able to elicit decreased activation of AKT.

**Figure 6:**
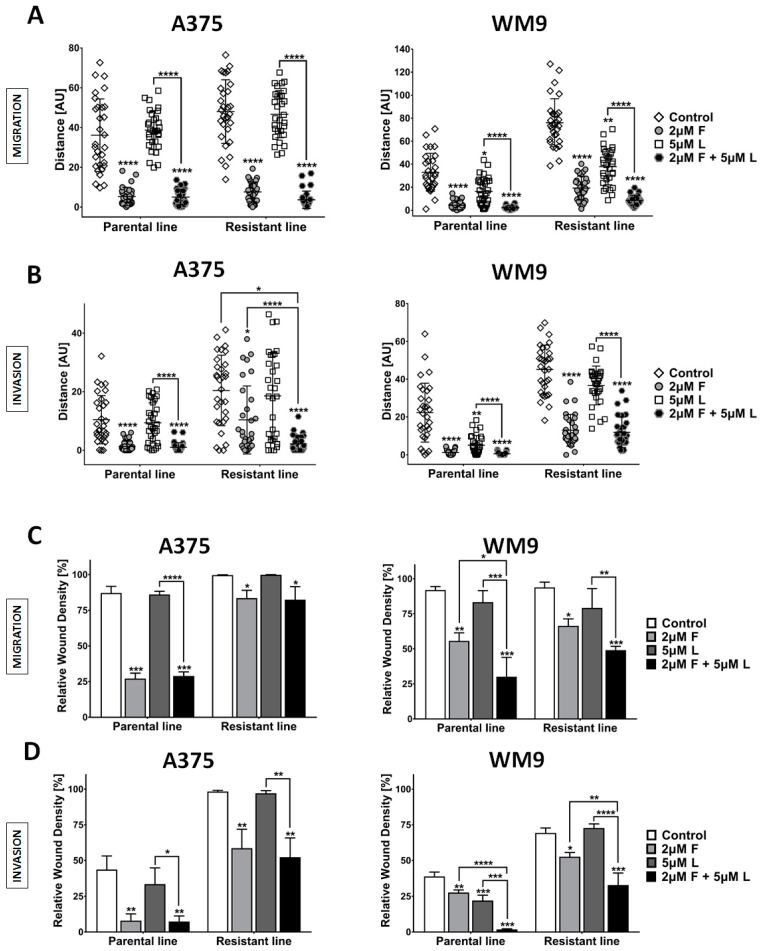
The effect of EGFR and MET inhibitors on the invasive abilities of resistant cells. Cells were seeded (**A**) exclusively on a layer of Matrigel or (**B**) were covered with an additional layer of the ECM on top of the cells and treated for 24 h with inhibitors of EGFR (L, lapatinib), and MET (F, foretinib) independently or as a pair at the indicated concentrations. (**A**,**B**) The inhibitors’ influence on cells’ spontaneous migration was evaluated using the IncuCyte Live-Cell Imaging System with an interval of 2 h. The results are presented as distances covered by cells in 24 h ± SD. At least 30 cells were quantified per repetition. The inhibitors’ impact on rates of migration (**C**) and invasion (**D**) of the examined cells was assessed using scratch wound assay. Quantification (**C**,**D**) of the results is presented as the mean relative wound density obtained from three independent experiments ± SD. Asterisks above the bars express significance vs. control unless indicated otherwise, with significance level set at * ≤0.05, ** ≤0.01, *** ≤0.001, **** ≤0.0001.

In the next step, we evaluated the influence of inhibitors on the invasive abilities of resistant cells, using analogous methods as previously described. While the parental lines were equally highly sensitive to the combination treatment and foretinib alone in both experimental setups, the resistant cells showed slightly different responsiveness, especially regarding the inhibition of spontaneous invasion (Figure 6A,B). The spontaneous migration of A375 RL was almost completely inhibited only after treatment with a duet of EGFR/MET inhibitors. In the case of WM9 RL, use of combination therapy produced a weaker response compared to the parental cell line. We also performed a wound healing assay following the treatment with inhibitors (Figure 6C,D). In both resistant cell lines, we were able to only partially inhibit migration and invasion; however, the use of foretinib alone and the pair of EGFR/MET inhibitors yielded the greatest effect, especially in the case of WM9 RL.

## 3. Discussion

Emerging drug resistance poses a great challenge on the way to full recovery from cancer. Its difficulty stems from the complexity of resistance mechanisms developed by cancer cells in response to long-term treatment. These mechanisms include a.o. genetic causes, epigenomic and transcriptomic changes, altered communication with the tumor microenvironment, an immunomodulatory effect, and the presence of CSCs [17]. Genetic mechanisms of resistance apply to BRAF alone: amplification of gene copying and alternative splicing, which were detected in 20%–32% of melanoma cases [18,19], or genes encoding proteins directly, and indirectly interacting with BRAF kinase. Mutations of NRAS, an upstream effector of BRAF, were found in 20% of melanoma patients and are considered to be mutually exclusive with BRAF mutations. They are able to not only reactivate the MAPK pathway, but are also thought to influence the PI3K/AKT pathway, which leads to lower incidence of apoptosis [20]. In the case of epigenomic or transcriptomic changes, which constitute ca. 40% of cases with identified BRAF mutation and treated with a specific inhibitor, many aberrations are present in elements of negative feedback loops regulating MAPK and PI3K/AKT pathways [17,21]. A great number of changes directly involve elements of the MAPK pathway—there is evidence of hyperactivation and overexpression of RAS or RTKs, e.g., IGFR1, PDGFRβ, EGFR [22,23,24]. In this study, we have focused on the evaluation of resistance mechanisms exhibited by human melanoma cells following long-term treatment with vemurafenib. Resistant cell lines established by us displayed an elevated level of the RTKs-EGFR and MET, on mRNA, and protein level, as well as their hyperactivation. Upregulation of EGFR signaling has also been reported in resistant A375 and Colo 829 cell lines [25]. Straussman et al. described increased activation and expression of MET and its ligand, HGF, in different melanoma cell lines treated with a progenitor of vemurafenib, PLX4720 [26]. Elevated levels of EGFR, MET, and PDGFRβ were also shown in several resistant cell lines generated from patients’ samples [27]. Additionally, cells with higher EGFR expression were more prone to the development of resistance to vemurafenib compared to low-EGFR cells [28].

Among other traits exhibited by cells resistant to inhibitor treatment is the emergence of CSCs, a distinctive population of malignant cells with the ability to self-renew and low sensitivity to standard treatment methods, which may confer drug resistance, and may be responsible for disease relapse or metastasis [29]. In the case of generated cell lines resistant to BRAF inhibitor, a changed cell morphology was distinctly noticeable. We observed that A375 RL and WM9 RL considerably differed in cell shape and cytoskeleton organization compared to parental cells—they displayed a spindle-like shape, were more spread, and had a greater number of actin stress fibers. We hypothesize that the upregulated level of vinculin, an integral component of focal adhesions, displayed by A375 and WM9 RL may be partially responsible for the observed phenotype. However, diverse reports can be found concerning the role of vinculin in cancer. Using human melanoma cell lines and a mouse model, Nelson et al. showed that the activation of vinculin was able to increase cell adhesion and sensitized cancer cells to treatment [30]. Meanwhile, Kawakami et al. detected vinculin along with integrin β1 in exosomes derived from prostate cancer cells, but their role is not clear [31]. Alterations in the morphology of cells treated with vemurafenib for 5–7 days were reported for a panel of human melanoma cells, where the flattened shape of cells was further connected to senescent phenotype [32]. An increase in stress fiber numbers was noticed in melanoma cells resistant to vemurafenib by Misek et al. [33], while a spindle-like or mesenchymal-like shape and elevated adhesion were reported in a number of studies on different cancer cells exhibiting drug resistance [34,35,36]. In addition to their altered shape, we also noticed that resistant lines showed a slightly lowered proliferation rate compared to parental ones. These two features are often associated with CSCs, which prompted us to check whether our resistant lines exhibit expression of CSC markers. In both cell lines, we found an increased level of CD44, a receptor of hyaluronic acid, which, upon ligand binding, can activate EGFR and ERBB2 [37], while only A375 RL demonstrated upregulation of CD166, also known as ALCAM (activated leukocyte cell adhesion molecule). The elevated expression of CD44, along with CD20, was earlier reported by Cordaro et al. in melanoma cells resistant to dabrafenib [38], while high levels of CD166 corresponded with poorer prognosis in patients suffering from uveal melanoma [39]. CSCs and cancer cells with acquired drug resistance often exhibit an increased level of invasive abilities, which results from hyperactivation of signaling pathways involving RTKs and PI3K/AKT.

In regard to the cancer cells’ interaction with their microenvironment, two major mechanisms are often mentioned: “therapy-induced secretome” and “senescence-associated secretory phenotype”, which consist of, for example, growth factors and MMPs that are able to transform neighboring normal cells to tumor-associated cells, or facilitate invasive abilities, respectively [32,40,41]. The A375 and WM9 resistant cell lines that we generated significantly differed from their parental counterparts in terms of motile abilities, both in the case of spontaneous migration/invasion and wound closure. Similar observations were described for both melanoma cell lines and cells derived from patients following long-term treatment with vemurafenib [42,43]. In a study by Zubrilov et al., resistant cells exhibited an elevated rate of transmigration through lung endothelial cells, which may facilitate the formation of metastases [44]. However, while migration is mostly dependent on the generation of protrusive force, which relies on focal adhesion turnover, invasion requires the activity of MMPs to form a pathway used by cells during movement through the dense ECM. While parental lines were already characterized by relatively high activity of gelatinases, visualized using fluorescently-labeled gelatin cover slips and gelatin zymography, the acquisition of drug resistance greatly increased the level of MMP activity, including MMP2, MMP9 and MMP14 (also known as MT1-MMP), which was further determined with gelatin zymography and a fluorimetric activity assay. Raised protein and mRNA levels of these MMPs with simultaneous downregulation of TIMP2 (tissue inhibitor of metalloproteinase 2) were also noticed by Sandri et al. [14], while Paulitschke et al. reported a heightened activation of MMP2 accompanied by decreased expression of TIMP3 [45], both in a model analogous to the one described in this work. The activation of MMP2 by MMP14, of which a high level in patients was correlated to poor prognosis, was also observed [41]. Additionally, Caporali et al. have shown increased secretion of MMP9 in A375 cells resistant to dabrafenib [46]. Interestingly, upregulation of MMP2 and MMP9 was linked with the depletion of nestin, a protein postulated as a melanoma stem cell marker [12,47], which was also observed in our research model.

In the literature, many promising therapies that are able to overcome drug resistance in mutant BRAF melanoma cells can be found. Aside from the most common treatment combining BRAF and MEK inhibitors (reviewed in [17]), there is a trend to look into drugs targeting other molecules involved in resistance mechanisms. Taking into account the overexpression and increased activation of RTKs in generated resistant cells, priming these molecules as potential therapy targets, we tested the efficiency of combination treatment using inhibitors of EGFR (lapatinib) and MET (foretinib) in this model. In our previous work, we have already demonstrated that dual inhibition of EGFR/MET was able to elicit a synergistic cytotoxic effect in melanoma cell lines, accompanied by a decrease in their invasive abilities [10,11]. Herein, we analyzed the effectiveness of a proposed therapy in A375 and WM9 cell lines resistant to vemurafenib, and noticed that both models demonstrate similar responsiveness to RTKs inhibitors compared with parental lines in terms of reduction of viability. The pair of drugs was able to significantly or completely abolish phosphorylation of EGFR, MET, and their downstream effectors, AKT and ERK, the effect of which was more pronounced in A375 RL. Moreover, combination treatment substantially diminished the ability of resistant cells to migrate and invade. However, we were not able to completely abolish the motile activity of cells exhibiting drug resistance. Other groups have also utilized inhibitors of EGFR combining them with BRAF-targeted drugs. Girotti et al. observed a significant reduction in the proliferation of melanoma cells *in vitro* and *in vivo* using gefitinib (EGFR and ERBB2 inhibitor) and PLX4720 (BRAF V600E inhibitor), as well as employing dasatinib (tyrosine kinase inhibitor with broad specificity) monotherapy [25]. Administration of a pan-ErbB inhibitor, canertinib, paired with vemurafenib also effectively decreased the growth of melanoma cells [48]. BRAF V600E mutation and drug resistance to BRAF inhibitors occurs also in colorectal cancer. Herr et al. were able to significantly diminish the proliferation and metabolic activity of colorectal cancer cells in vitro combining various BRAF inhibitors with dual blockers of EGFR and ERBB2, like lapatinib [49]. An interesting case of a patient with BRAF mutant colorectal cancer was reported by Pietrantonio et al. [50]. The patient was initially treated with vemurafenib and an anti-EGFR drug, but over time developed resistance mediated by MET upregulation. He was then subjected to combination treatment including vemurafenib with crizotinib, a MET and ALK inhibitor, which resulted in the restoration of sensitivity to therapy. This case seems to validate the administration of anti-cancer agents that are able to target more RTKs simultaneously. However, considering the fact that a high percentage of patients exhibiting drug resistance display hyperactivation of the PI3K/AKT pathway, utilizing inhibitors of PI3K or AKT seems valid. Promising results were reported by Atefi et al. and Sweetlove et al., who paired BRAF or MEK inhibitors with drugs blocking PI3K, AKT or its downstream effector, mTOR [51,52].

Finally, it is worth taking into account the differences between the two examined cell lines used to generate the resistance cells. A great majority of the observed features may be a result of cells’ origin—the A375 cell line is derived from a primary amelanotic melanoma, while the WM9 cell line was obtained from melanoma metastasis to lymph nodes. As we have shown in this work, A375 initially displayed greater sensitivity to vemurafenib treatment, and, after acquisition of resistance, presented a more distinct phenotype compared to the WM9 cell line and its resistant counterpart. We presume that the cell line derived from metastasis originally demonstrates high aggressiveness (low sensitivity to inhibitor treatment, a high level of MMP2), while primary cells show greater potential to change, which is seen in almost all tested features.

Our results support the validity of using combination therapy directed against RTKs overexpressed and hyperactivated in melanoma cells resistant to treatment with inhibitors of BRAF V600E. Here, we have demonstrated a comprehensive characterization of a drug-resistant model including various mechanisms of resistance, such as upregulation of up- and downstream effectors of BRAF, acquisition of cancer stem-like traits, and elevated invasive and proteolytic activities. Additionally, our findings provide proof that dual inhibition of EGFR and MET in resistant cells not only reduces cell viability but is also able to diminish the invasiveness of drug-resistant cells.

## 4. Materials and Methods

### 4.1. Cell Culture

Human melanoma cell lines derived from a primary tumor (A375) and metastasis to lymph nodes (WM9) were acquired from ATCC (American Type Culture Collection, Manassas, VA, USA), and Rockland Immunochemicals, Inc. (Limerick, Ireland), respectively. Cells were maintained in full DMEM medium (4.5 g/L glucose, 1.5 g/mL NaCO_3_, 4 mM glutamine, 110 mg/mL sodium pyruvate) (IITD PAN, Wroclaw, Poland) containing 1% antibiotic-antimycotic mixture (penicillin/streptomycin/amphotericin B) (ThermoFisher Scientific, Waltham, MA, USA) and 10% fetal bovine serum (FBS) (Gibco). Cells were passaged twice a week using trypsin (IITD PAN, Wroclaw, Poland) and standard 25 cm^2^ culture flasks, and maintained at 37 °C, 5% CO_2_.

To obtain the resistant lines, cells were cultured in the presence of vemurafenib (Santa Cruz Biotechnologies, Dallas, TX, USA), an inhibitor of BRAF V600E, starting from a concentration of 0.05 µM and ending at 3.2 µM for A375 or 8 µM for WM9, the final concentration depending on the overall sensitivity of both treated cell lines. After the cells achieved the resistance to the inhibitor, they were maintained in a culture medium with 1 µM of vemurafenib to prevent the loss of resistance.

### 4.2. Braf V600E Mutation Verification

To verify the mutation status of BRAF kinase in examined cells, total RNA was isolated using the GeneMATRIX Universal RNA Purification Kit (EURx, Gdansk, Poland) following the manufacturer’s instructions. After DNase I (EURx, Gdansk, Poland) treatment, reverse transcription reaction was performed using 0.5 μg of RNA and the High Capacity cDNA Reverse Transcription Kit (Applied Biosystems, Foster City, CA, USA) according to the manufacturer’s protocol. The BRAF encoding sequence was amplified in a PCR reaction with primers forward: 5’ATGGCGGCGCTGAGCG3’ and reverse: 5’CGAAATCCTTGGTCTCTAATCAA3’, where cDNA from melanoma cell lines served as a template. Next, after agarose gel electrophoresis, the separated PCR product was isolated from gel with the GeneElute Gel Extraction Kit (Sigma-Aldrich, Saint Louis, MO, USA) and analyzed by a sequencing service (Genomed S.A., Warsaw, Poland).

### 4.3. Cytotoxicity Evaluation

Cell Proliferation Kit II (XTT, Roche, Basel, Switzerland) was used to determine cell viability and cell proliferation rate. First, 96-well culture plates were coated with 1 mg/mL Matrigel (BD Biosciences, San Jose, CA, USA) for 1 h at 37 °C, followed by seeding 10,000 (A375) or 8,000 (WM9) cells, which then were allowed to grow for 24 h. In the case of the 3D viability test, an additional layer of Matrigel was deposited on top of the cells, and allowed to polymeraze for 1 h at 37 °C. Then, growth medium was replaced with full medium containing 0.1–50 µM of vemurafenib or the growth factors EGF (5 nM) (BD Biosciences, San Jose, CA, USA) and HGF (30 ng/mL) (Sigma-Aldrich, Saint Louis, MO, USA), with inhibitors of EGFR (lapatinib, 5 µM) and MET (foretinib, 2 µM) used independently or as a pair. Control cells were exposed to 0.1% DMSO (drugs’ solvent). After 24 h, the medium was replaced with a fresh one with an added XTT (2,3-Bis-(2-Methoxy-4-Nitro-5-Sulfophenyl)-2H-Tetrazolium-5-Carboxanilide) mixture, and incubated for 3 h at 37 °C. Colorimetric signal was measured at 450 nm with a µQuant microplate spectrophotometer (Bio-Tek Instruments, Inc., Winooski, VT, USA) and Gen5 software (ver. 2.05, Bio-Tek Instruments, Inc., Winooski, VT, USA). Acquired results were background corrected, and calculated to obtain the viability (%) and proliferation rate of the examined cells (fold change), where an additional plate measured at the time of inhibitor administration was used (t0) [53]. Additionally, IC50 values, which represent the concentration at which a substance exerts half of its maximal inhibitory effect, were calculated for all four cell lines following vemurafenib treatment, using the online tool Quest Graph™ IC50 Calculator (AAT Bioquest, Inc., Sunnyvale, CA, USA) [54].

### 4.4. Western Blot Analysis

Cells were seeded in 60 mm Petri culture dishes and allowed to grow for 24 h. Then, culture medium was replaced with fresh full medium without additional components or containing growth factors, and foretinib and/or lapatinib, in previously indicated concentrations. After 4 h (with inhibitors) or 24 h (characterization of cell lines) of incubation, cells were placed on ice, washed with PBS and harvested in lysis buffer (50 mM TRIS-HCl pH 7.4, 5% SDS, 8.6% saccharide, 0.45% urea, 1 mM dithiothreitol), followed by centrifugation for 10 min at 12,000 rpm and 4 °C. Supernatants were transferred to fresh tubes, and protein content was measured by the standard Bradford method [55]. Samples containing 10 µg of protein were separated using SDS-PAGE electrophoresis [56], followed by transfer to nitrocellulose sheets. The quality of transfer was determined with Ponceau S staining. Then, membranes were blocked with 5% non-fat milk in TBST for 1 h at RT and probed overnight at 4 °C with primary antibodies directed against the following: EGFR, MET, GAPDH (glyceraldehyde 3-phosphate dehydrogenase), pAKT1/2/3 (S473), ALCAM and nestin (Santa Cruz Biotechnologies, Dallas, TX, USA); pEGFR (Y1068), pMET (Y1234/1235), and pERK (T202/Y204) (Cell Signaling Technology Inc., Danvers, MA, USA); CD44 (DSHB, Iowa City, IA, USA), and vinculin (Bio-Rad, Hercules, CA, USA), followed by a 1 h incubation with secondary antibodies conjugated with horseradish peroxidase directed against primary mouse and rabbit antibodies (Cell Signaling Technology Inc., Danvers, MA, USA). Next, membranes were incubated with ECL substrate (Bio-Rad, Hercules, CA, USA), and the chemiluminescent signal was acquired with ChemiDoc (Bio-Rad, Hercules, CA, USA), using ImageLab software (ver. 6.0, Bio-Rad, Hercules, CA, USA).

### 4.5. Confocal Microscopy Imaging

Glass cover slips were placed in 24-well culture plates and coated with 1 mg/mL Matrigel (BD Biosciences, San Jose, CA, USA) for 1 h at 37 °C. Cells were seeded on prepared slides and after 24 h were fixed with 4% formaldehyde, followed by permeabilization with 0.1% Triton X-100. Next, cover slips were blocked with 1% bovine albumin for 1 h at RT and then probed overnight with primary rabbit antibodies directed against cortactin (Santa Cruz Biotechnologies, Dallas, TX, USA) at 4 °C. After several washing steps, slides were probed with secondary antibodies conjugated with Alexa 488 directed against rabbit antibodies (ThermoFisher Scientific, Waltham, MA, USA) and stained with phalloidin conjugated with Alexa 568 (ThermoFisher Scientific, Waltham, MA, USA), Hoechst 33342 to visualize F-actin, and DNA, respectively, for 1 h at RT. Finally, after washing, cover slips were mounted with Dako fluorescent mounting medium (Dako, Agilent, Santa Clara, CA, USA) on microscope slides and imaged with Leica SP8 (Leica, Wetzlar, Germany) using LAS X software (ver. 3.3.0, Leica, Wetzlar, Germany).

### 4.6. qPCR

To evaluate the expression level of MET and EGFR in tested cells, total RNA was isolated with the GeneMATRIX Universal RNA Purification Kit (EURx, Gdansk, Poland) according to the manufacturer’s instructions. Following DNase I (EURx, Gdansk, Poland) treatment, a reverse transcription reaction was performed using 0.5 μg of RNA and the High Capacity cDNA Reverse Transcription Kit (Applied Biosystems, Foster City, CA, USA) according to the manufacturer’s protocol. Quantitative PCR was executed using StepOne Plus Real-Time PCR (Applied Biosystems, Foster City, CA, USA) in a mixture consisting of TaqMan^®^ Universal Master Mix II (Applied Biosystems, Foster City, CA, USA), specific probes and 10 ng of cDNA in a total volume of 10 μL. The following TaqMan^®^ probes were used: GAPDH (Hs02758991-g1), MET (Hs01565576-m1) and EGFR (Hs01076091-m1) (Applied Biosystems, Foster City, CA, USA). GAPDH served as a housekeeping gene. Based on the comparative CT (threshold cycle value) method (ΔCT = CT gene of interest − CT housekeeping gene), the relative quantification of gene expression was calculated. Three independent experiments were performed for all cell lines.

### 4.7. Scratch Wound Assay

First, 96-well IncuCyte ImageLock plates (Essen BioScience, Ann Arbor, Michigan, USA) were coated with 1 mg/mL Matrigel (BD Biosciences, San Jose, CA, USA), followed by seeding 50,000 cells per well. After 24 h, when cells reached confluency, standardized wounds were formed with Wound Maker (Essen BioScience, Ann Arbor, Michigan, USA), and then detached cells were washed out with warm culture medium. For the invasion assay, an additional layer of Matrigel was deposited on top of the cells and allowed to polymerize for 1 h at 37 °C. Next, a fresh medium without additives or containing growth factors and inhibitors of EGFR and/or MET in previously indicated concentrations, was added to the wells, and the plates were placed in an IncuCyte Live-Cell Analysis System (Essen BioScience, Ann Arbor, Michigan, USA) for 24 h, with images acquired every 2 h with IncuCyte ZOOM software (ver. 2018A, Essen BioScience, Ann Arbor, Michigan, USA). Relative wound density was calculated using an IncuCyte Cell Migration Software module (Essen BioScience, Ann Arbor, Michigan, USA). The experiment was performed in triplicate and each repetition consisted of 3 replicates.

### 4.8. Spontaneous Migration Assay

IncuCyte ImageLock plates with 96 wells (Essen BioScience, Ann Arbor, Michigan, USA) were coated with 1 mg/mL Matrigel (BD Biosciences, San Jose, CA, USA), followed by seeding of 1000 cells per well. The next day, the culture medium was replaced with a fresh one without additives or containing previously mentioned concentrations of growth factors and foretinib and/or lapatinib. In the case of invasion, assay cells were covered with an additional layer of 1 mg/mL Matrigel and placed in the incubator to allow the matrix to polymerize, and then the medium was deposited on top of the ECM. The plate was placed in an IncuCyte Live-Cell Analysis System (Essen BioScience, Ann Arbor, Michigan, USA) for 24 h, with images acquired every 2 h with IncuCyte ZOOM software (ver. 2018A, Essen BioScience, Ann Arbor, Michigan, USA). Distances covered by cells and their trajectories were measured with the ImageJ Manual Tracking plugin (developed by Fabrice Cordeli, Institute Curie, Orsay, France). Additionally, representative images showing cell morphology were acquired.

### 4.9. Gelatin-FITC Degradation Assay

Glass cover slips precoated with poly-L-lysine were deposited in 24-well cell culture plates and washed with PBS. To quench reactive side chains, 0.5% glutaraldehyde was added for 15 min. After washing with PBS, cover slips were coated with gelatin-FITC (Invitrogen, Waltham, MA, USA) for 10 min, followed by inactivation of residual glutaraldehyde with 5 mg/mL sodium borohydrate for 1 min and two washing steps. Then, cells were seeded on top of the cover slips and allowed to grow and digest gelatin for 18 h. Afterwards, cells were fixed with 4% formaldehyde and stained with phalloidin conjugated with Alexa 568 to visualize F-actin. Dark spots on a green fluorescent background indicated the areas of digestion. Images were acquired with an Olympus FV500 confocal laser scanning microscope (Olympus, Tokio, Japan) and FluoView software (Olympus, Tokio, Japan). 

### 4.10. Gelatin Zymography

Conditioned media from cells grown in serum-free media for 72 h were collected and centrifuged for 10 min at 1500 rpm and 4 °C. Supernatants were concentrated using Amicon Ultra-4 centrifugal filters (Merck Millipore, Burlington, MA, USA), and protein content was evaluated with the standard Bradford procedure [55]. Samples were prepared using 3 µg of protein and non-reducing sample buffer, while gels for electrophoresis contained 0.1% gelatin. After electrophoresis, gels were washed with buffer containing Triton X-100 to remove the SDS and then incubated in detergent-free buffer for 12 h at 37 °C. Gels were then stained with Coomassie Brilliant Blue R for 30 min, followed by destaining to visualize areas of digestion seen as transparent spots on a blue background. Images of gels were captured with ChemiDoc (Bio-Rad, Hercules, CA, USA) and, after inversion of colors, the densitometry was performed using ImageLab software (ver. 6.0, Bio-Rad, Hercules, CA, USA). Obtained results were calculated as a fold change of MMP activity between the parental and resistant lines.

### 4.11. MMP14 Activity Assay

To evaluate MMP14 activity, the SensoLyte 520 MMP14 Assay Kit (AnaSpec, Fremont, CA, USA) was used according to the manufacturer’s protocol. Briefly, cells were seeded in 60 mm Petri dishes and washed with PBS after 24 h, and then collected in assay buffer. Lysates were centrifuged at 4 °C for 10 min at 2500× *g*, then supernatants were transferred to fresh tubes. The protein content was determined with the standard Bradford method [55], followed by preparation of samples, each containing 30 µg of protein. To activate the MMP14, samples were incubated for 2 h at 37 °C in the presence of 1 nM of APMA (4-aminophenylmercuric acetate). Then, MMP14 substrate was added to start an enzymatic reaction, and, after 30 min of incubation at 37 °C, the reaction was stopped with a stop solution. Recombinant MMP14 (Merck Millipore, Burlington, MA, USA) was used as a positive control. The fluorescence of digested substrate was measured at 490/520 nm using Infinite M1000 Pro (Tecan, Männedorf, Switzerland) and i-control software (Tecan, Männedorf, Switzerland). The results were background corrected and calculated as a fold change of activity between the parental and resistant lines.

### 4.12. Statistical Analysis

All experiments were performed in triplicate and tested with a Mann–Whitney test (spontaneous migration) or two-tailed Student’s *t*-test (other experiments) to show differences between the parental and resistant lines, or with Kruskal–Wallis test (spontaneous migration) and one-way ANOVA followed by Bonferroni post hoc test (the rest of experiments) to determine differences between treatment conditions with inhibitors or different time points in the wound healing assay. Statistical analysis was conducted using GraphPad Prism software (ver. 7.05, San Diego, CA, USA).

## Supplementary Materials

Supplementary materials can be found at https://www.mdpi.com/1422-0067/21/1/113/s1. Figure S1. Evaluation of sensitivity of examined parental (PL) and resistant (RL) cells to vemurafenib treatment. (A) Dose-response graphs and (B) IC50 values were calculated using Quest Graph™ IC50 Calculator, based on data obtained in XTT assay. Figure S2. Characterization of resistant cells. Morphology of parental (PL) and resistant (RL) cells seeded on Matrigel-coated culture plates visualized with IncuCyte Live-Imaging System using their High Definition (HD) Phase-Contrast Imaging Mode. Scale bar is set at 300 μm.
