# Supplementary material for: Characterization of Melanoma Cell Lines Resistant to Vemurafenib and Evaluation of Their Responsiveness to EGFR- and MET-Inhibitor Treatment
Source: Int J Mol Sci. 2019 Dec 23;21(1):113. doi: 10.3390/ijms21010113 (PMC6981576; doi:10.3390/ijms21010113)
Supplement: Supplementary file 1 [file ijms-21-00113-s001.pdf]

SUPPLEMENTARY FILE

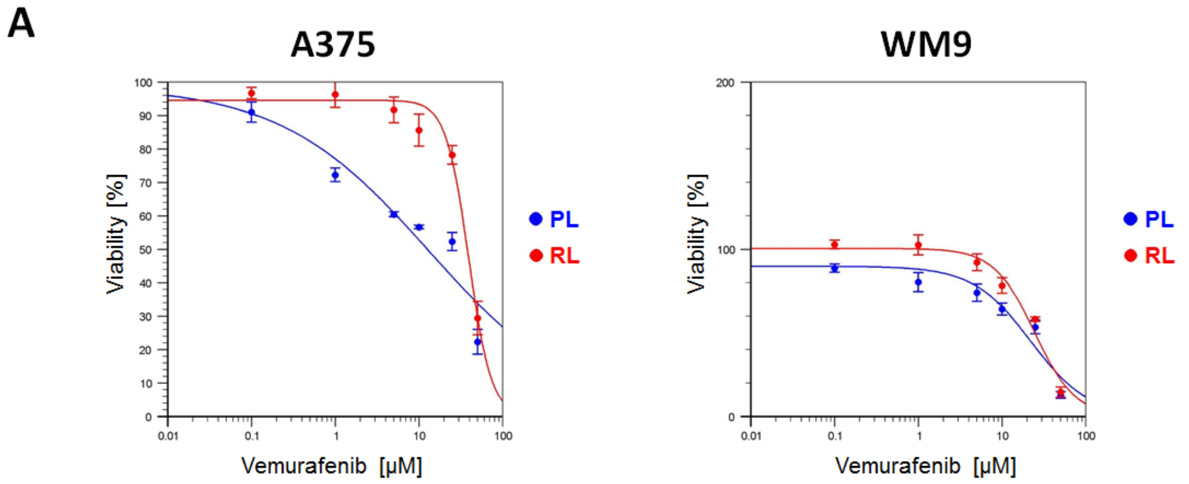

B

	A375 PL	A375 RL	WM9 PL	WM9 RL
IC 50 [μM]	13.217	39.378	22.103	24.581

Figure S1. Evaluation of sensitivity of examined parental (PL) and resistant (RL) cells to vemurafenib treatment. (**A**) Dose-response graphs and (**B**) IC50 values were calculated using Quest Graph™ IC50 Calculator, based on data obtained in XTT assay.

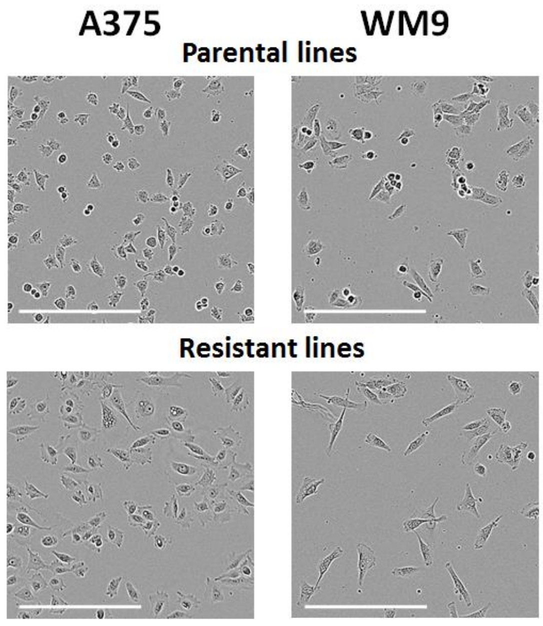

Figure S2. Characterization of resistant cells**.** Morphology of parental (PL) and resistant (RL) cells seeded on Matrigel-coated culture plates visualized with IncuCyte Live-Imaging System using their High Definition (HD) Phase-Contrast Imaging Mode. Scale bar is set at 300 μm.
